# Regularized binormal ROC method in disease classification using microarray data

**DOI:** 10.1186/1471-2105-7-253

**Published:** 2006-05-09

**Authors:** Shuangge Ma, Xiao Song, Jian Huang

**Affiliations:** 1 Department of Biostatistics, University of Washington, Seattle, WA 98195, USA; 2 Department of Statistics & Actuarial Science and Program in Public Health Genetics, University of Iowa, Iowa City, IA 52242, USA

## Abstract

**Background:**

An important application of microarrays is to discover genomic biomarkers, among tens of thousands of genes assayed, for disease diagnosis and prognosis. Thus it is of interest to develop efficient statistical methods that can simultaneously identify important biomarkers from such high-throughput genomic data and construct appropriate classification rules. It is also of interest to develop methods for evaluation of classification performance and ranking of identified biomarkers.

**Results:**

The ROC (receiver operating characteristic) technique has been widely used in disease classification with low dimensional biomarkers. Compared with the empirical ROC approach, the binormal ROC is computationally more affordable and robust in small sample size cases. We propose using the binormal AUC (area under the ROC curve) as the objective function for two-sample classification, and the scaled threshold gradient directed regularization method for regularized estimation and biomarker selection. Tuning parameter selection is based on *V*-fold cross validation. We develop Monte Carlo based methods for evaluating the stability of individual biomarkers and overall prediction performance. Extensive simulation studies show that the proposed approach can generate parsimonious models with excellent classification and prediction performance, under most simulated scenarios including model mis-specification. Application of the method to two cancer studies shows that the identified genes are reasonably stable with satisfactory prediction performance and biologically sound implications. The overall classification performance is satisfactory, with small classification errors and large AUCs.

**Conclusion:**

In comparison to existing methods, the proposed approach is computationally more affordable without losing the optimality possessed by the standard ROC method.

## Background

Microarray experiments that monitor gene expression profiles associated with different disease phenotypes have become commonplace in biomedical research. Each DNA sequence represented in microarrays can be considered a potential biomarker. Classification and prediction using such genomic measurements may contribute to a better understanding of the genetic pathways involved in diseases, and hence may lead to better diagnosis of disease and better prescription of treatment. Analysis of genomic data is challenging due to high dimensionality of data and low sample size. Although the number of genes assayed is large, but there may be only a small number of genes that are associated with variations of phenotypes. By employing standard methods directly, we usually obtain estimates that are not "regular", i.e., estimates are not unique or ill-behaved. Regularization, through which we achieve unique and well-behaved estimates, is usually needed. Regularization can be achieved via model reduction or variable selection methods.

Several dimension reduction techniques have been employed for classification using genomic data. Examples include the partial least squares [1], the principal component regression [2], and the singular value decomposition under the Bayesian framework [3,4], among others. By using low dimensional projections of covariates as features in model estimation, one may obtain estimators with better prediction performance due to the bias-variance tradeoff. One drawback of such dimension reduction techniques is that all genes are used in estimation and prediction. Biological interpretation of such classifiers are usually not straightforward. Moreover, if certain genes are not associated with the clinical outcome, it is important to exclude them from the predictive model.

An alternative approach to the dimension reduction techniques is to use methods that are capable of simultaneous biomarker selection and model fitting, which can be realized by penalization. Such methods include the least absolute shrinkage and selection operator-LASSO [5], the least angle regression-LARS [6], and the threshold gradient directed regularization method-TGDR [7]. These methods can produce parsimonious models with a small number of biomarkers and hence more lucent biological interpretations.

In this article, we propose an approach for simultaneous estimation and biomarker selection using a scaled TGDR method.

It is important to assess both false-positive and false-negative errors, since the clinical and financial consequences of the two types of errors can differ significantly. A common practice is to use the receiver operating characteristic (ROC) curve [8,9], where the classification performance can be measured by the area under the ROC curve (AUC). Advantages of the ROC method include: (1) it does not assume a parametric form of the class probability. This is different from the logistic regression method. Although one can construct an ROC curve from logistic regression, it assumes a parametric form of the class probability. This parametric assumption may be not be satisfied; (2) it is adaptable to outcome-dependent samplings, for example the case control design; and (3) it is capable of penalizing false positives and false negatives differently. Therefore, the ROC method may be preferable in biomarker selection and classification using genomic measurements.

Pepe [10] proposed the empirical AUC as the objective function for combining multiple biomarkers in a low dimensional setting. Ma and Huang [11] proposed a smooth sigmoid approximation of the empirical AUC for high-dimensional data. An alternative to the empirical AUC is the binormal AUC [8]. The binormal AUC technique was developed parallel to, but separated from the empirical AUC method. For small sample sizes, the empirical AUC may change dramatically due to small perturbations and differ significantly from the expected AUC, whereas the binormal AUC is more stable. Studies with low-dimensional biomarkers show that the binormal AUC may provide valuable information beyond the empirical AUC [8]. For data with high dimensional covariates or large sample sizes, the binormal AUC is computationally much more affordable than the empirical AUC. Since both the empirical AUC and the binormal AUC are extensively used in low dimensional settings, it is of great interest to extend the study of [11] and explore use of the binormal AUC for disease classification with microarray data.

In this article, we proposed an approach for biomarker selection and classification with microarray data by optimizing the binormal AUC. The scaled TGDR method, which is a modified version of the TGDR, is adopted for estimation and biomarker selection. Tuning parameters are selected using *V*-fold cross validation, and Monte Carlo based methods are proposed for evaluation purposes. We assess the proposed approach by extensive simulation studies and demonstrate it on two cancer datasets. Comparing to the method in [11], which uses a smoothed version of the empirical AUC as the objective function, the contributions of this paper are as follows. First, the binormal AUC, which is at least as important as the empirical AUC, is used as the objective function. Second, we propose using the scaled TGDR, which can significantly reduce the computational cost. Moreover, the occurrence index, which is a way to rank the selected biomarkers and measure their relative stability in the presence of sampling variation, is proposed in this paper.

## Results

### Simulation studies

Simulation studies are used to examine the finite sample performance of the proposed regularized ROC method. In simulation study I, we look at the case where the genes are assumed to be independently normally distributed. This setting satisfies all the assumptions for the binormal ROC method [8] and the underlying model is the logistic regression model. In simulation study II, we consider independent genes with marginal distributions other than the normal. The corresponding ROCs are still binormal since the linear predictors for both the diseased and non-diseased groups are normally distributed after a common monotone transformation. We investigate the performance of the proposed method when the transformation is intentionally neglected and the marginal normality assumption is violated. In simulation study III, we study the effect of correlations among genes. In simulation study IV, we investigate the case where marginal normality cannot be obtained with simple transformations. The simulation settings are related to but much more comprehensive than those in [11].

Using simulation settings similar to those in [12], we first generate *d *= 1000 dimensional vectors from the diseased and non-diseased populations. We consider the following sample size combinations (*n*_*D*_, *n*_*H*_) = (15, 15), (10, 20), (50, 50) and (30, 70), where *n*_*D *_and *n*_*H *_denote the number of samples in the groups with *Y *= 1(diseased) and *Y *= 0 (non-diseased), respectively. Different marginal and joint distributions of the genes are considered, representing a variety of data structures encountered in real medical studies. We assume a model in which a fraction *π *of the potential biomarkers are differentially expressed and hence associated with the binary outcome of interest.

#### Simulation study I

All the 1000 biomarkers are assumed to be independent and normally distributed with variance 1. We consider *π *= 0.01, *π *= 0.05 and *π *= 0.25, which represent the cases where a small number, a moderate number and a large number of biomarkers are associated with the outcome. Moreover, we investigate the following three scenarios:

1. (Small effect) There is a shift of 0.5 in the mean expression levels for the differentially expressed biomarkers;

2. (Moderate effect) The fold change is 1.5 unit difference in mean;

3. (Large effect) A shift of 5 units in the mean is considered.

The underlying model is the logistic regression model with regression coefficients equal to two times the mean shifts for genes differentially expressed and zero for the rest.

For each simulated dataset, we first randomly sample a training set of size 2/3 × (*n*_*D *_+ *n*_*H*_) without consideration of the stratification of the binary outcome. The testing set is composed of the rest observations. We construct the proposed ROC estimate based on the training set only. Tuning parameters are selected using 3-fold cross validation. The predictive classification error and binormal AUC are computed for the testing set based on the estimate obtained from the training set. Summary statistics based on 200 simulated datasets are given in Table 1.

**Table 1:** Simulation study I. Means of AUC and classification error (with their standard errors in parentheses) for small, moderate and large mean differences.

		*π *= 0.01			*π *= 0.05			*π *= 0.25		
(*n*_*H*_, *n*_*D*_)		small	moderate	large	small	moderate	large	small	moderate	large
(15, 15)	AUC	0.63(0.18)	0.88(0.10)	0.99(0.01)	0.64(0.17)	0.92(0.09)	1.00(0.00)	0.71(0.17)	0.95(0.07)	1.00(0.00)
	Error	0.37(0.16)	0.16(0.16)	0.00(0.00)	0.37(0.17)	0.12(0.13)	0.00(0.00)	0.32(0.15)	0.07(0.10)	0.00(0.00)
(20, 10)	AUC	0.58(0.18)	0.86(0.12)	0.99(0.01)	0.65(0.18)	0.92(0.08)	1.00(0.00)	0.66(0.18)	0.95(0.06)	1.00(0.00)
	Error	0.37(0.17)	0.17(0.14)	0.00(0.00)	0.34(0.15)	0.11(0.13)	0.00(0.00)	0.32(0.17)	0.07(0.10)	0.00(0.00)
(50, 50)	AUC	0.66(0.09)	0.96(0.03)	1.00(0.00)	0.72(0.09)	0.99(0.01)	1.00(0.00)	0.77(0.09)	0.99(0.01)	1.00(0.00)
	Error	0.36(0.09)	0.09(0.06)	0.00(0.00)	0.33(0.08)	0.04(0.04)	0.00(0.00)	0.29(0.08)	0.04(0.04)	0.00(0.00)
(70, 30)	AUC	0.66(0.12)	0.95(0.03)	1.00(0.00)	0.70(0.10)	0.98(0.02)	1.00(0.00)	0.77(0.09)	0.99(0.01)	1.00(0.00)
	Error	0.32(0.09)	0.09(0.05)	0.00(0.00)	0.29(0.08)	0.05(0.04)	0.00(0.00)	0.24(0.08)	0.03(0.04)	0.00(0.00)

It can be seen that for moderate and large effect cases, the binormal AUCs are close to 1 with very small classification errors. The classification performance for small mean shift cases is less satisfactory, although still acceptable when the sample size is not too small, due to the relatively small signal to noise ratios. As sample size increases, the classification performance improves. The classification performance also improves with more differentially expressed genes. The empirical AUCs are very close to the binormal AUCs. Results for the empirical AUCs are omitted here.

#### Simulation study II

We consider data settings similar to those in Simulation I, with *π *= 0.05 and 1.5 unit shift in mean for differentially expressed genes. We assume all biomarkers are independent and marginally distributed as Uniform[-$\sqrt{3}$, $\sqrt{3}$] Gamma(l/4,1/2), and 2$\sqrt{2}$Beta(0.5, 0.5) for the non-diseased group. The diseased group has the same marginal distributions with only mean shifts. All three marginal distributions have variance 1 and represent three different distribution scenarios: uniformly distributed gene expressions; gene expressions with a skewed unimodal distribution (Gamma); and gene expressions clustering around the upper and lower limits (Beta). Proper transformations can lead to marginal normal distributions. We intentionally neglect the transformations and employ the proposed approach directly. The purpose is to investigate the performance of the proposed approach when marginal distribution assumption is violated.

Summary statistics based on 200 simulated datasets are shown in Table 2. For comparison, we also reproduce the results from the normal distribution case in Simulation I. It can be seen that the proposed approach has satisfactory classification performance under all scenarios. The effect of different marginal distributions is not significant. The empirical AUCs are also reasonably close to the binormal AUCs. Simulation studies with different mean shifts and different choices of *π *lead to conclusions similar to those for simulation study I. Those results are omitted here.

**Table 2:** Simulation study II. Means of AUC and classification error (with their standard errors in parentheses) for *π *= 0.05 and moderate mean differences. Marginal distributions: *Normal*(0, 1), *Uniform*[-$\sqrt{3}$, $\sqrt{3}$] *Gamma*(1/4, 1/2) and 2$\sqrt{2}$*Beta*(0.5, 0.5).

(*n*_*H*_, *n*_*D*_)		Normal	Uniform	Gamma	Beta
(15, 15)	AUC	0.92(0.09)	0.92(0.08)	0.89(0.12)	0.92(0.08)
	Error	0.12(0.13)	0.12(0.13)	0.10(0.12)	0.13(0.14)
(20, 10)	AUC	0.92(0.08)	0.92(0.08)	0.89(0.12)	0.92(0.08)
	Error	0.11(0.13)	0.13(0.12)	0.13(0.13)	0.13(0.13)
(50, 50)	AUC	0.99(0.01)	0.99(0.01)	0.95(0.06)	0.99(0.01)
	Error	0.04(0.04)	0.04(0.04)	0.06(0.06)	0.03(0.04)
(70, 30)	AUC	0.98(0.02)	0.98(0.01)	0.96(0.06)	0.99(0.01)
	Error	0.05(0.04)	0.04(0.04)	0.07(0.06)	0.03(0.04)

#### Simulation study III

In simulation studies I and II, possible correlations among genes have been neglected. In simulation study III, we investigate the case where the genes are correlated. We still assume *π *= 0.05 and 1.5 unit shift in mean for differentially expressed genes. For the non-diseased subjects, let *Z*_*i*1_, *Z*_*i*2_, ..., *i *= 1, ..., *n*_*H*_, be a sequence of i.i.d. standard normal random variables. Denote the *d *× *n*_*H *_dimensional gene expression matrix for healthy subjects as *X*. For the *j*^*th *^gene of the *i*^*th *^subject, we consider the following models:

1. Model 1: $X_{ij}=H\left(\sum_{l=\left(j-1\right)\times m+1}^{\left(j-1\right)\times m+k}Z_{il}/\sqrt{k}\right)$ with *k *= 10, *m *= 7; *H *= $\sqrt{3}$(2Φ - 1), where Φ is the cumulative distribution for the standard normal.

2. Model 2: Same as Model 1, but with *k *= 10, *m *= 3.

3. Model 3: Same as Model 1, with *H *as the identify function.

4. Model 4: Same as Model 2, with *H *as the identify function.

This simulation setting has been considered in [13]. For models 1 and 2, the genes are marginally uniformly distributed. For models 3 and 4, the genes are marginally normally distributed. For genes in models 1 and 3, the correlations between all genes (differentially and non-differentially expressed) are weak; whereas models 2 and 4 are examples of strongly correlated genes. Note that we not only assume differential genes are correlated, we also assume differentially expressed and non-differentially expressed genes are correlated.

Summary statistics based on 200 replicates are shown in Table 3. For comparison, we also reproduce the results with independently, marginally uniformly (normally) distributed genes. We can see from Table 3 that the effects of correlation are negligible. We looked at other correlation structures and obtained similar results. Moreover the effect of different marginal distributions are not significant.

**Table 3:** Simulation study III. Means of AUC and classification error (with their standard errors in parentheses) for *π *= 0.05 and moderate mean differences. Marginal distributions: *Uniform*[-$\sqrt{3}$, $\sqrt{3}$] and *Normal*(0, 1). Independent, weakly correlated and strongly correlated genes.

		Uniform			Normal		
(*n*_*H*_, *n*_*D*_)		Independent	Weak	Strong	Independent	Weak	Strong
(15, 15)	AUC	0.92(0.08)	0.91(0.08)	0.91(0.09)	0.92(0.09)	0.91(0.09)	0.90(0.10)
	Error	0.12(0.13)	0.14(0.13)	0.14(0.13)	0.12(0.13)	0.12(0.12)	0.14(0.12)
(20, 10)	AUC	0.92(0.08)	0.91(0.10)	0.91(0.08)	0.92(0.08)	0.91(0.09)	0.89(0.10)
	Error	0.13(0.12)	0.12(0.13)	0.13(0.13)	0.11(0.13)	0.13(0.13)	0.13(0.12)
(50, 50)	AUC	0.99(0.01)	0.98(0.01)	0.98(0.02)	0.99(0.01)	0.98(0.02)	0.98(0.02)
	Error	0.04(0.04)	0.05(0.04)	0.06(0.04)	0.04(0.04)	0.05(0.05)	0.07(0.05)
(70, 30)	AUC	0.98(0.01)	0.98(0.02)	0.97(0.02)	0.98(0.02)	0.98(0.02)	0.97(0.03)
	Error	0.04(0.04)	0.04(0.04)	0.06(0.05)	0.05(0.04)	0.05(0.05)	0.07(0.05)

#### Simulation study IV

The binormal AUC is theoretically constructed based on the joint normal distribution assumption on the linear combination of the covariates in the diseased and non-diseased population after a common monotone transformation. We now consider the case when marginal normality cannot be achieved with simple transformation. Specifically, we investigate the case when the marginal gene distributions for the diseased class and the non-diseased class are different. We assume the number of genes is 1000 and there are 50 subjects in each group. We assume that *π *= 0.05 percent genes are differentially expressed. Moreover, we assume the genes in the non-diseased group are independent and have standard normal distribution. For subjects in the diseased class, we assume the genes are independently distributed with:

1. Scenario 1: Uniform [1.5 - $\sqrt{3}$, 1.5 + $\sqrt{3}$] for differential genes and Uniform [-$\sqrt{3}$, $\sqrt{3}$]otherwise;

2. Scenario 2: 2$\sqrt{2}$Beta(0.5, 0.5) - $\sqrt{2}$ + 1.5 for differential genes and 2$\sqrt{2}$Beta(0.5,0.5) - $\sqrt{2}$ otherwise;

3. Scenario 3: Gamma(l/4, 1/2) + 1 for differential genes and Gamma(l/4, 1/2) - 0.5 otherwise.

Under all three scenarios, the differentially expressed genes for the diseased subjects have mean 1.5 and variance 1, and non-differential genes have mean 0 and variance 1. Based on 200 replicates, the mean AUCs are 0.99 (0.01), 0.99 (0.01) and 0.98 (0.04) under scenarios 1–3, respectively, where the values in the parentheses are the corresponding standard errors. The mean classification errors are 0.05 (0.04), 0.04 (0.04) and 0.07 (0.05), respectively.

### Colon and estrogen data

#### Data description

##### Colon data

In this dataset, expression levels of 40 tumor and 22 normal colon tissues for 6500 human genes are measured using the Affymetrix gene chip. A selection of 2000 genes with the highest minimal intensity across the samples has been made by [14], and these data are publicly available at [15]. The colon data have been analyzed in several previous studies using other statistical approaches, see for example [1,11,16-18].

##### Estrogen data

This dataset was first presented by [3,4]. It includes expression values of 7129 genes of 49 breast tumor samples. The expression data were obtained using the Affymetrix gene chip technology and are available at [19]. The response describes the status of the estrogen receptor (ER). Among the 49 samples, 25 are positive (ER+) and 24 are negative (ER-). We threshold the raw data with a floor of 100 and a ceiling of 16000. Genes with max(*expression*)/min(*expression*) < 10 and/or max(*expression*) - min(*expression*) < 1000 are also excluded [20]. 3332 (46.7%) genes pass the first step screening. A base 2 logarithmic transformation is then applied. The estrogen data have also been studied by [11,16].

#### Estimation

The ROC estimate is identifiable only up to a scale constant [8]. So prior to the analysis, we need to identify the "anchor biomarker," i.e, the gene whose estimated coefficient will be set as a constant for identifiability purposes. We select the anchor biomarker as follows. Compute the sample standard errors of the *d *biomarkers *se*_(1)_, ..., *se*(*d*) and denote their median as *med.se*. Compute the adjusted standard errors as 0.5(*se*_(1) _+ *med.se*), ..., 0.5(se_(*d*) _+ *med.se*). Then the biomarkers are ranked based on the *t*-statistics computed with the adjusted standard errors. This adjusted *t*-statistic is similar to a simple shrinkage method discussed in [21]. It has been observed in microarray studies that the standard t-statistic can be very large even if there is very little difference between the mean expressions (for the diseased and healthy classes), due to the small variance estimate caused by small sample size [13]. So compared with the standard t-statistic, the adjusted t-statistic takes into account variability as well as fold change. The biomarker with the largest absolute value of the adjusted *t*-statistic is chosen as the anchor biomarker. For the anchor biomarker, if the sample mean of the diseased class is larger, *β*_(1) _= 1.0, otherwise *β*_(1) _= -1.0.

Further investigation of the genes that have passed the first step screening shows that quite a few of those genes have very little variations across sample. Including such genes in the joint modeling may create unreliable estimates. Moreover, it is unlikely all the 2000 genes for the colon data (3332 for the estrogen data) will be associated with the outcome. So we only use the 500 genes with the largest absolute values of the adjusted *t*-statistics for classification. The genes are then standardized to have zero means and unit variances. The same anchor marker detection and pre-processing have been considered in [11] and are shown to be well-behaved.

Note that there is no computational limitation on how many genes can be used in the ROC classification. Empirical studies in [13] show that the performance of certain statistical approaches decreases dramatically as the number of genes increases. So it is believed that identification of the top 500 genes will help to generate more reliable estimate.

We use three-fold cross validation for tuning parameter selection. We show in Table 4 the number of genes with nonzero coefficients and the CV scores for each fixed threshold value *τ*. It can be seen that generally the number of nonzero coefficients decreases as *τ *increases. However, the change of CV score is very small. Our extensive empirical studies show that in general a larger *τ *will lead to a more parsimonious model. Parsimonious models are preferred when the CV scores are comparable. So we choose *τ *= 1.0 for both datasets.

**Table 4:** Colon and estrogen data. Model features for different *τ*. Variable: number of genes with nonzero coefficients.

	Colon		Estrogen	
*τ*	variable	CV	variable	CV
1.0	18	2.64	20	2.93
0.9	26	2.61	27	2.93
0.8	73	2.63	79	2.93
0.7	278	2.70	139	2.93
0.6	453	2.74	307	2.94
0.5	481	2.76	422	2.94
0.4	492	2.79	490	2.96
0.3	498	2.81	499	2.97
0.2	500	2.76	500	2.97
0.1	500	2.74	500	2.97
0.0	500	2.74	500	2.97

In the TGDR, variable selection is achieved by thresholding some estimated coefficients to be exactly zero based on their derivatives, so the corresponding covariates are not selected in the final model. The genes in Tables 5 and 6 have nonzero coefficients and other genes have estimated coefficients zero and hence not selected. Since the gene expressions have been normalized to have unit variances, the estimated coefficients are directly comparable. Larger absolute values of coefficients indicate stronger influences.

**Table 5:** Colon Data: genes with nonzero coeficients.

GeneID	*β*	Gene Description
Hsa.949	0.15	M59807, NATURAL KILLER CELLS PROTEIN 4 PRECURSOR.
Hsa.8219	-1.01	R46753, CYCLIN-DEPENDENT KINASE INHIBITOR 1 (Homo sapiens).
Hsa.10047	0.10	T51849, TYROSINE-PROTEIN KINASE RECEPTOR ELK PRECURSOR.
Hsa.8214	0.40	R62549, PUTATIVE SERINE/THREONINE-PROTEIN KINASE B0464.5.
Hsa.8175	-0.31	H49870, MAD PROTEIN (Homo sapiens).
Hsa.2483	-0.10	D14665, Human mRNA for ORF, complete cds.
Hsa.3016	1.33	T47377, S-100P PROTEIN (HUMAN).
Hsa.5392	0.15	T62947, 60S RIBOSOMAL PROTEIN L24 (Arabidopsis thaliana).
Has.341	-0.71	M26683, Human interferon gamma treatment inducible mRNA.
Hsa.1410	1.00	R54097, TRANSLATIONAL INITIATION FACTOR 2 BETA SUBUNIT (HUMAN).
Hsa.2928	0.54	X63629, H.sapiens mRNA for p cadherin.
Hsa.9246	-0.40	T47383, ALKALINE PHOSPHATASE, PLACENTAL TYPE 1 PRECURSOR.
Hsa.1240	-0.30	M31994, Human cytosolic aldehyde dehydrogenase (ALDH1) gene, exon 13.
Hsa.1454	-0.96	M82919, Human gamma amino butyric acid receptor beta-3 subunit mRNA.
Hsa.627	1.00	M26383 Human monocyte-derived neutrophil-activating protein (MONAP) mRNA.
Hsa.2688	0.25	X60489, Human mRNA for elongation factor-1-beta.
Hsa.6814	0.51	H08393, COLLAGEN ALPHA 2(XI) CHAIN (Homo sapiens).
Hsa.1491	0.94	M35531, Human GDP-L-fucose:beta-D-galactoside 2-alpha-l-fucosyltransferase mRNA.

**Table 6:** Estrogen data: genes with nonzero coeficients.

GeneID	*β*	Gene Description
AB002365_at	0.06	AB002365, Human mRNA for KIAA0367 gene, partial cds.
D43772_at	-0.10	D43772, Human squamous cell carcinama of esophagus mRNA for GRB-7 SH2 domain protein.
D87468_at	-0.61	D87468, Human mRNA for KIAA0278 gene, partial cds.
HG2755-HT2862_at	0.10	T-Plastin.
J02871_s_at	0.35	J02871, Human lung cytochrome P450 (IV subfamily) BI protein, complete cds.
K02054_at	0.55	K02054, Human gastrin-releasing peptide mRNA, complete cds.
K03460_at	-0.06	K03460, Human alpha-tubulin isotype H2-alpha gene, last exon.
M11718 _at	0.05	M11718, Human alpha-2 type V collagen gene, 3' end.
M24069_at	0.25	M24069, Human DNA-binding protein A (dbpA) gene, 3' end.
M32053_at	0.45	M32053, Human H19 RNA gene, complete cds (spliced in silico).
M81758_at	-0.10	M81758, Homo sapiens skeletal muscle voltage-dependent sodium channel alpha subunit (SkM1) mRNA.
M83186_at	0.05	M83186, Human cytochrome c oxidase subunit VIIa (COX7A) muscle isoform mRNA, complete cds.
U01062_at	-0.20	U01062, Human type 3 inositol 1,4,5-trisphosphate receptor (ITPR3) mRNA, complete cds.
U03057_at	0.15	U03057, Human actin bundling protein (HSN) mRNA, complete cds.
U28386_at	0.05	U28386, Human nuclear localization sequence receptor hSRP1alpha mRNA, complete cds.
U60115_at	0.05	U60115, Human skeletal muscle LIM-protein SLIM1 mRNA, complete cds.
U82169_at	-0.40	U82169, Human frizzled homolog (FZD3) mRNA, complete cds.
X03635_at	1.00	X03635, Human mRNA for oestrogen receptor.
X56667_at	-0.20	X56667, Human mRNA for calretinin.
X86693_at	0.10	X86693, H.sapiens mRNA for hevin like protein.

More detailed information about these genes can be found on the NCBI website. For example, for the colon data, Hsa.949 is from the interleukin 32 gene (IK32). This gene encodes a member of the cytokine family. Expression of this protein is increased after the activation of T-cells by mitogens or the activation of NK cells by IL-2. This protein induces the production of TNFalpha from macrophage cells. Hsa.8219 (gene symbol: CDKN1A) encodes a potent cyclin-dependent kinase inhibitor. The encoded protein binds to and inhibits the activity of cyclin-CDK2 or -CDK4 complexes, and thus functions as a regulator of cell cycle progression at Gl. The expression of this gene is tightly controlled by the tumor suppressor protein p53, through which this protein mediates the p53-dependent cell cycle G l phase arrest in response to a variety of stress stimuli. This protein can interact with proliferating cell nuclear antigen (PCNA), a DNA polymerase accessory factor, and plays a regulatory role in S phase DNA replication and DNA damage repair. Hsa.627 encodes a protein that is a member of the CXC chemokine family. This chemokine is one of the major mediators of the inflammatory response and is secreted by several cell types. It functions as a chemoattractant, and is also a potent angiogenic factor. This gene is believed to play a role in the pathogenesis of bronchiolitis, a common respiratory tract disease caused by viral infection. This gene and the other ten members of the CXC chemokine gene family form a chemokine gene cluster in a region mapped to chromosome 4q.

For the estrogen data, D43772 (gene symbol: GRB7) encodes a protein belonging to a small family of adapter proteins that are known to interact with a number of receptor tyrosine kinases and signaling molecules. This gene encodes a growth factor receptor-binding protein that interacts with epidermal growth factor receptor (EGFR) and ephrin receptors. The protein plays a role in the integrin signaling pathway and cell migration by binding with focal adhesion kinase (FAK). K02054 (gene symbol: GRP) encodes a member of the bombesin-like family of gastrin-releasing peptides. Its preproprotein, following cleavage of a signal peptide, is further processed to produce either the 27 aa gastrin-releasing peptide or the 10 aa neuromedin C. These smaller peptides regulate numerous functions of the gastrointestinal and central nervous systems, including release of gastrointestinal hormones, smooth muscle cell contraction, and epithelial cell proliferation. These peptides are also likely to play a role in human cancers of the lung, colon, stomach, pancreas, breast, and prostate. U03057 (gene symbol: FSCN1) plays a role in a number of diseases. For example, it impacts the progression of hormone receptor-negative breast cancer. It is also expressed in cutaneous CD30+ lymphoproliferative disorders and is a candidate marker of disease progression. X03635 (gene symbol: ESR1) is the estrogen receptor (ESR) that is a ligand-activated transcription factor composed of several domains important for hormone binding, DNA binding, and activation of transcription.

#### Evaluation

We first randomly partition the data into training and testing sets. We then identify the top 500 genes with the largest adjusted t-statistics based on the training sets only. So the 500 genes used in each individual partition and computation may be different, although there is considerable overlap.

**Figure 1:**
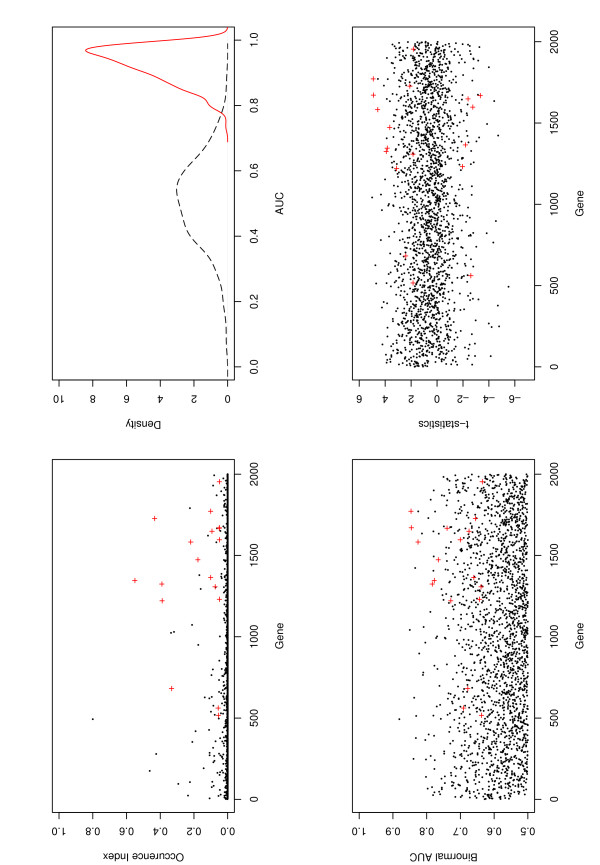
**Colon data**. X axis: natural order of genes. Left-upper panel: occurrence index, red "+": genes identified with the TGDR. Right-upper panel: kernel density estimation of the OPD (solid line) and PPD (dashed line) of AUG. Left-lower panel: Binormal AUC of every gene. Lower-right panel: t-statistics of every gene.

**Figure 2:**
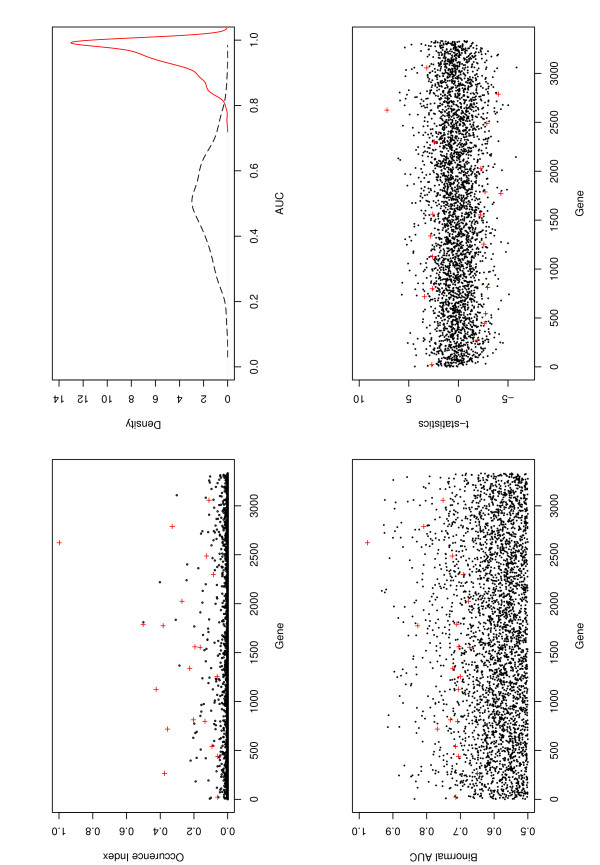
**Estrogen data**. X axis: natural order of genes. Left-lower panel: occurrence index, red "+": genes identified with the TGDR. Right-lower panel: kernel density estimation of the OPD (solid line) and PPD (dashed line) of AUC. Left-lower panel: Binormal AUC of every gene. Lower-right panel: t-statistics of every gene.

The occurrence index, which measures the relative stability of individual selected genes in the joint model, is shown in Figures 1 and 2 for the colon and estrogen data, respectively. We observe that many genes selected with the proposed approach have relatively high occurrence index compared to genes not selected, which suggests that the ROC estimates are relatively stable for these two specific datasets. On the other hand, we also see that the occurrence index, even for the genes selected, can be low in absolute terms. Most selected genes have occurrence index less than 0.50. Similar phenomenon has been observed in [22]. The genes identified in the final model do not necessarily have the largest occurrence index. For example, in the colon data the gene with the highest occurrence index was not selected in the model. This is not surprising since (a) the objective function is the binormal AUC and the genes were selected to optimize this objective function; (b) the genes were selected based on all the available data, whereas the occurrence index was calculated using partitioned data. The occurrence index is one way to evaluate the relative importance of the genes after they are selected and it provides a measure of stability of the genes in the presence of sampling variation, but it does not measure the prediction power of the genes. Furthermore, the genes significantly associated with the outcome in different subsets are not necessarily important when the whole dataset is used. Thus we do not select genes based on the occurrence index.

The classification performance of the proposed method for the two datasets is evaluated using the Monte Carlo approach described in the Methods section. Both the number of random partitions for the observed data and the number of permutations are 1000. For the colon data, the ROC estimate has mean AUCs 0.94 (0.05) and 0.50 (0.13) for the OPD and PPD, respectively, where the values in the parentheses are the standard errors. Corresponding mean classification errors are 0.14 (0.06) for observed data and 0.44 (0.11) for randomly permuted data, respectively. In the upper-right panel of Figures 1 and 2, we also show the kernel density estimates of the OPD and PPD of the AUC. We can see that the two distributions are well separated. A simple Wilcoxon test of the difference of these two distributions gives p-value < 0.0001. For the same dataset and using the top ranked 200 genes based on the marginal Wilcoxon rank test, Table 1 in [16] shows that the mean classification errors are 0.16 (LogitBoost, 100 iterations), 0.18 (AdaBoost), 0.18 (1-nearest-neighbor) and 0.15 (classification tree). In [17], 2000 genes are used and the SVM based methods have mean AUCs 0.85 and mean classification errors 0.18, whereas the principal component analysis based approaches have even smaller AUCs and larger classification errors. Using the same set of 500 genes, the logistic-LASSO method in [11] has mean AUCs 0.88 and mean classification errors 0.08; the SMRC method in [11] has mean AUCs 0.91 and mean classification errors 0.14. So for the colon data, the proposed binormal ROC method performs better than other approaches in terms of AUC and classification error, although the improvement is not dramatic since most other approaches behave reasonably well. We also note that since different sets of genes are used in [16,17], the above results can only provide a rough comparison.

For the estrogen data, the proposed ROC estimate has mean AUCs 0.95 (0.05) and 0.50 (0.14) for the OPD and PPD, respectively, with corresponding mean classification errors 0.08 (0.06) and 0.48 (0.12). For the same dataset, Dettling and Buhlmann (2003, Table 1) use 200 genes selected based on the marginal Wilcoxon tests and yield classification errors 0.04, 0.06 and 0.08 with different LogitBoost methods, classification error 0.04 for AdaBoost, 0.14 for 1-nearest-neighbor approach and 0.04 by using the classification tree. Using the same set of genes, the logistic-LASSO [11] has mean AUCs 0.92 and mean classification errors 0.12; whereas the SMRC [11] has mean AUCs 0.96 and mean classification errors 0.06.

Note that in our study, joint models are considered, i.e, we consider the joint effects instead of marginal effects of genes. Identification of differentially expressed genes associated with clinical outcomes based on marginal significance has been well investigated and accepted. So as a simple evaluation, we show the marginal binormal AUC and t-statistic for each individual gene in Figures 1 and 2. Similar evaluation has been considered in [23] as a gene ranking method. We can see from Figures 1 and 2 that several genes we identify do have large marginal binormal AUCs and t-statistics; both up- and down- regulated genes are identified in the final models. However, we also identify some genes with small binormal AUCs or small t-statistics. It is believed that these genes are important in the joint models, but not in the marginal models. Variable selection using TGDR looks at all the variables and considers the relative importance of each variable in terms of its contribution to change in the objective function, but is not based on marginal significance. So the results do not necessarily correspond to those based on marginal significance.

**Figure 3:**
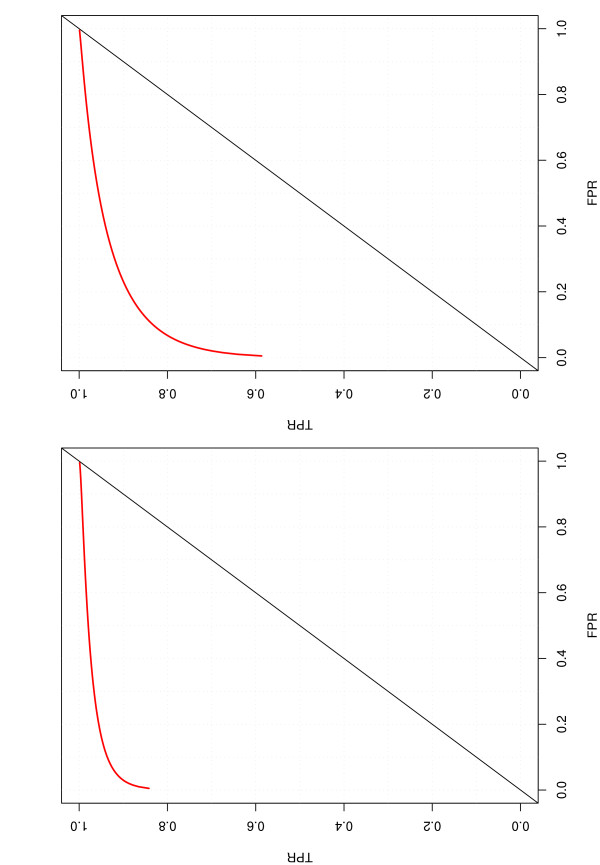
**Binormal ROC plot**. Left panel: training set. Right panel: testing set.

When binormal ROC method is used, it is of interest to examine whether the ROC curve is concave. Pepe [8] pointed out the binormal ROC is not concave when the variances of the two groups are not equal. However, she also pointed that "Swets (1986) and Hanley (1988, 1996) conclude that the binomal ROC curve provides a good approximation to a wide range of ROC curves that occur in practice [24-26]. This may be because the undesirable behavior of the approximating binormal ROC usually occurs over a very small part of the ROC curve." As a simple check, we show in Figure 3 the plots of binormal ROC for a randomly selected training set and a testing set. We can clearly see the concavity of the binormal ROC. Examination of other training and testing sets shows similar results.

We note that although the genes identified using the two datasets exhibit strong predictive power for disease statuses as demonstrated above, the analysis here does not provide information on whether they are just genomic markers correlated with the disease statuses or are actually in the pathways leading to the diseases. Indeed, as is typical with microarray results, further investigation based on independent assays and/or independent samples is desirable.

## Conclusion

The ROC method has been successfully used for disease classification using low- dimensional biomarkers. The empirical AUC method has been successfully implemented in [11]. In this article, we propose using the binormal AUC, which is a useful alternative to the empirical AUC, as the objective function for disease classification using high dimensional genomic data. The scaled TGDR is employed for regularization and biomarker selection. Extensive simulation studies and applications to two cancer data show that the proposed approach can generate satisfactory classification results with a small number of identified biomarkers with biologically sound implications. Although theoretically the binormal AUC is constructed under the normal distribution assumption, the proposed approach still behaves satisfactorily when this assumption is seriously violated. Beyond the merits carried by the ROC method, the proposed approach has the advantage of little computational cost. Using the scaled gradient significantly reduces the computational complexity. It takes only a few seconds to analyze dataset with 1000 genes and 100 subjects using R [27] code, whereas the empirical AUC method takes several minutes or longer.

In our data analyses, we selected 500 genes with the largest adjusted t-statistics. The proposed approach has no limitation on the number of genes that can be used in the final model. We note that use of 500 genes is somewhat subjective. Same analyses with 200 genes and all the genes have also been carried out (results not shown), and there are considerable overlaps among genes identified. We prefer using 500 genes because this set of genes should include most (if not all) important genes without including too much noise. We used the same datasets as in [11]. Although both methods are ROC based and the same sets of genes are used, the identified biomarkers are different. Considering that both the empirical AUC and the binormal AUC methods are extensively used, such difference warrants further investigation and confirmation by independent studies.

In our study, the adjusted t-statistic is used instead of the standard t-statistic. The reason we used the adjusted t-statistic is that the unadjusted t-statistics can be large for genes with small mean differences in expression levels, if the standard error is very small. Because of a large number of genes on each array, there will usually be genes with very small standard errors, and some of these genes will have small means as well. This phenomenon has been discussed by [28] in motivating their adjusted t-statistic for significance analysis of microarrays (SAM). Similar variance adjust has also been considered in [29]. However, a recent study [30] points out that such variance adjust may not be necessary. Since current study is not conclusive, we use the adjusted t-statistic and leave the choice of t-statistic versus adjusted t-statistic to a future study.

In this paper, we proposed Monte Carlo based methods for evaluation of prediction and stability of individual genes. The proposed methods are closely related to cross validation and permutation methods. The problem of evaluation is extremely important for high dimensional classification, but it has not been extensively studied. Although the proposed methods have sound, intuitive implications, further studies of their theoretical properties would be of interest.

We focus on the linear combination of genes that maximizes the binormal-AUG. In [31], linear combinations of genes favoring certain specificity or other criterion are investigated. If maximization of specificity is of interest, we can then combine Liu's methods with certain variable selection techniques, for example LASSO or the proposed TGDR. We leave this to a future study. We only discussed two-sample classification in this article. When extension of the binormal AUC to multi-class study exists, the proposed estimation and regularization method can be used. We postpone multi-class study to a future report.

## Methods

### Regularized ROC classification

#### Stochastic model

Consider a study with *n *subjects, where the outcome *Y *is a binary random variable with the values 0 and 1 denoting the absence and presence of disease, respectively. For the *i*^*th*^subject, expression values of *d *genes, X_*i *_= (X_*i*,1_, ..., X_*i*,*d*_), are measured. When using the ROC classification, we predict the phenotype *Y *based on the "linear risk scores" *β*'X, where *β *= (*β*_(1)_, ..., *β*_(*d*)_) is a *d*-dimensional vector of regression parameters and *β*' denotes its transpose. For example, we classify *Y *= 1 if *β*'X > *c*, for a cutoff *c *chosen based on the estimated ROC, otherwise *Y *= 0. When a semiparametric single index model *P*(*Y *= 1|X) = *G*(*β*'X) is assumed, the ROC classification is optimal [10]. Here *G *is an unspecified increasing link function. Note that when the single index model assumption is violated, the ROC method, although no longer optimal, still provides meaningful classification results [8,10].

#### ROC method

To evaluate the performance of a classifier based on the linear risk scores *β*'X with different choices of *β*, we employ the widely used measurements of classification accuracy in medicine, namely the true and false positive rates (TPR and FPR). Also known as sensitivity and 1-specificity, respectively, TPR and FPR are defined as

TPR(c) = P(*β'*X ≥ *c*|*Y *= 1) and FPR(c) = P(*β'*X ≥ *c*|*Y *= 0),

for any cutoff *c*. The TPR and FPR can be summarized by the ROC curve, which is a two-dimensional plot of {(FPR(*c*), TPR(*c*)) : -∞ <*c *< ∞}. The ROC curve demonstrates the balance between the true positive and false positive rates. Classification rules that have (*FPR*(*c*),*TPR*(*c*)) close to (0,1) indicate satisfactory discriminators, while those with (*FPR*(*c*), *TPR*(*c*) near the 45° line cannot discriminate between the diseased and non-diseased classes.

For the *n *subjects, denote ⅅ and ℍ as the index sets for the diseased and non-diseased subjects with sizes *n*_*D *_and *n*_*H*_, respectively. Let X^*D *^denote the biomarkers of a diseased subject and X^*H *^the biomarkers of a non-diseased subject. The overall performance of a classifier can be measured by the AUC, with larger AUC indicating better performance. For any linear risk score *β'*X, the empirical AUC is

${\text{AUC}}_{empirical}\left(\beta \right)=\frac{1}{n_{D}n_{H}}\sum_{i\in D,j\in ℍ}I\left(\beta^{\prime }X_{i}-\beta^{\prime }X_{j}>0\right),\;\left(1\right)$

where *I*(·) is the indicator function. Pepe [10] proposes to maximize (1) to obtain an optimal linear risk score. [11] extends Pepe's work to high dimensional settings by considering a Sigmoid approximation of the empirical AUC.

Alternatively, we consider the binormal AUC. Assume that X^*D *^and X^*H*^, after a common monotone transformation, have normal distributions X^*D *^~ *N*(*μ*^*D*^, Σ^*D*^) and X^*H *^~ *N*(*μ*^*H*^, Σ^*H*^), respectively. Under this so-called binormal model, the AUC can be written as [8]

${\text{AUC}}_{normal}\left(\beta \right)=\Phi \left(\frac{\beta^{\prime }(\mu^{D}-\mu^{H})}{{\left(\beta^{\prime }(\Sigma^{Η}+\Sigma^{D})\beta \right)}^{1/2}}\right),\;\left(2\right)$

where Φ is the normal distribution function. For a sample with *n *subjects, the binormal AUC can be estimated by

${\hat{\text{AUC}}}_{normal}\left(\beta \right)=\Phi \left(\frac{\beta^{\prime }({\hat{\mu }}^{D}-{\hat{\mu }}^{H})}{{\left(\beta^{\prime }({\hat{\Sigma }}^{Η}+{\hat{\Sigma }}^{D})\beta \right)}^{1/2}}\right),\;\left(3\right)$

where $\hat{\mu }$ and $\hat{\Sigma }$ denote the sample mean and the sample variance-covariance matrix, respectively. We propose using (3) as the objective function for two-sample classification from microarray data. Define the ROC estimator of *β *as:

$\hat{\beta }=\arg\max_{\beta }{\hat{\text{AUC}}}_{normal}\left(\beta \right).\;\left(4\right)$

We note that this estimator is only identifiable up to a scale constant. Without loss of generality, we assume |*β*_(1)_| = 1.0, where *β*_(1) _denotes the first component of *β*, i.e, the first biomarker is the "anchor biomarker." We suggest a simple way of determining the anchor biomarker in the Results section.

The binormal AUC (2) is obtained under the normal distribution assumption. As pointed out by Pepe [8], "the binormal ROC curve plays a central role in ROC analysis. In much the same way that the normal distribution is a classic model for distribution functions, the binormal ROC curve is a classic model for ROC curves." When the normal distribution assumption does not hold, the objective function (3) still generates a meaningful estimate, i.e, using the linear combination of genes that maximizes classification power as measured by the AUC. For reference, see [8]. Simulation studies under model mis-specification are provided in the Results section.

#### Regularized estimation

Maximization of the binormal AUC with low dimensional covariates has been investigated in [31]. With large sample size and a small number of covariates, the maximizer in [31] is unique and well-defined and variable selection is not of interest. However with the microarray data, there exist multiple maximizers of the binormal AUC; moreover, variable selection is needed along with estimation. So regularized estimation, through which unique and stable estimates are generated, is needed. In addition, the following regularization method provides a way of variable selection.

For simplicity of notation and without loss of generality, we assume that the first biomarker is the anchor and *β*_(1) _= 0.1, and we still use *β *to denote the remaining coefficients (*β*_(2)_, ..., *β*_(*d*)_)'. The TGDR approach was originally proposed for the linear regression [7]. This method first establishes a parameter path in the high dimensional coefficient space using the gradient descent method, and then identifies the best model along the parameter path with certain cross validation techniques. Here we adapted this method to the binormal AUC. Let *β*(*v*) denote the parameter path indexed by *v *∈ [0, ∞). Let Δ*v *be the infinitesimal positive increment as in ordinary gradient descent methods [7]. In implementation of this algorithm, we choose Δ*v *= 1 × 10^-4^. For any threshold 0 ≤ *τ *≤ 1, the TGDR algorithm consists of the following iterative steps:

1. Initialize *β*(0) = 0 and *v *= 0.

2. Compute the *relative gradient *function *g*(*v*), where *g*(*v*) = -*∂*${\hat{\text{AUC}}}_{normal}$(*β*)/*∂β *× *s*(*v*) and *s*(*v*) is a positive scale function depending on the data and *v *Denote the *j*^*th *^component of *g*(*v*) as *g*_*j*_(*v*). If max_*j*_{|*g*_*j*_(*v*)|} = 0, stop the iterations. Otherwise, *g*(*v*) = *g*(*v*)/max_*j*_{|*g*_*j*_(*v*)|}.

3. Compute the vector *f*(*v*) of length *d*, where the *j*^*th *^component of *f*(*v*) is *f*_*j*_(*v*) = *I*{|*g*_*j*_(*v*)| ≥ *τ*·max_*l*_|*g*_*l*_(*v*)|}.

4. Update *β*(*v *+ Δ*v*) = *β*(*v*) + Δ*v *× *g*(*v*) × *f*(*v*) and replace *v *by *v *+ Δ*v*, where the product of *f *and *g *is component-wise.

5. Steps 2–4 are repeated *k *times. The number of iterations *k *is determined by cross validation as described below.

When max_*l*_{|*g*_*l*_(*v*)|} is less than a pre-specified criterion, the iteration can be stopped. We recommend tracking the magnitude of max_*l*_{|*g*_*l*_(*v*)|} and the plot of the cross validation function as a function of *k *to determine the number of iterations in step 5.

The proposed TGDR has been adapted and modified from [7], in the sense that the scaled, relative gradient is used instead of the gradient function in step 2. This modification is equivalent to using the original gradient, but different Δ*v*, at each iteration. So the modified TGDR can still lead to complete parameter paths, as long as Δ*v *is small enough. We compute the relative gradient *g*(*v*) as follows. First note:

$\begin{array}{l} \partial {\hat{\text{AUC}}}_{normal}\left(\beta \right)/\partial \beta =\varphi \left(\frac{\beta^{\prime }({\hat{\mu }}^{D}-{\hat{\mu }}^{H})}{{\left(\beta^{\prime }({\hat{\Sigma }}^{H}+{\hat{\Sigma }}^{D})\beta \right)}^{1/2}}\right)\times {\left(\beta^{\prime }({\hat{\Sigma }}^{H}+{\hat{\Sigma }}^{D})\beta \right)}^{-3/2}\\ \times \;\left\{\left({\hat{\mu }}^{D}-{\hat{\mu }}^{H}{)}^{\prime }(\beta^{\prime }({\hat{\Sigma }}^{H}+{\hat{\Sigma }}^{D})\beta )-\beta^{\prime }({\hat{\mu }}^{D}-{\hat{\mu }}^{H})\beta ({\hat{\Sigma }}^{H}+{\hat{\Sigma }}^{D}\right)\right\}, \end{array}$

where *ϕ *is the normal density function. Set $s\left(v\right)={\left\{\varphi \left(\frac{\beta^{\prime }({\hat{\mu }}^{D}-{\hat{\mu }}^{H})}{{\left(\beta^{\prime }({\hat{\Sigma }}^{H}+{\hat{\Sigma }}^{D})\beta \right)}^{1/2}}\right)\times {\left(\beta^{\prime }({\hat{\Sigma }}^{H}+{\hat{\Sigma }}^{D})\beta \right)}^{-3/2}\right\}}^{-1}$. Note that *s*(*v*) is positive as long as *β *has nonzero components. The relative gradient

$g\left(v\right)=-\left\{\left({\hat{\mu }}^{D}-{\hat{\mu }}^{H}{)}^{\prime }(\beta^{\prime }({\hat{\Sigma }}^{H}+{\hat{\Sigma }}^{D})\beta )-\beta^{\prime }({\hat{\mu }}^{D}-{\hat{\mu }}^{H})\beta ({\hat{\Sigma }}^{H}+{\hat{\Sigma }}^{D}\right)\right\}$

has less computational cost compared to the original gradient. Although the modification is not dramatic, it can reduce the computational time from minutes to seconds with high dimensional data.

Detailed discussions of the TGDR algorithm can be found in [7] where a graphic presentation is also available (Figures 1 and 3 therein). The tuning parameters *τ *and *k *jointly determine the property of $\hat{\beta }$. Stable estimates are expected with non-zero *τ*, since *τ *guarantees that only covariates with large gradients are included in the model. Loosely speaking *k *measures convergence and more importantly the complexity of the model: for a fixed *τ*, smaller *k *leads to more parsimonious models. Specifically when *τ *≈ 0, $\hat{\beta }$ is dense for all values of *k*. When *τ *≈ 1, $\hat{\beta }$ is sparse for small *k *and remains so for a relatively large number of iterations, but will become dense eventually. At the extreme when *τ *= 1, the TGDR usually increases in the direction of a single covariate in each iteration. This mimics the incremental forward stage-wise strategy described in [32]. When *τ *is in the middle range, the characteristics of $\hat{\beta }$ are between those for *τ *= 0 and *τ *= 1. The threshold parameter *τ *is constrained in the interval [0, 1]. Unfortunately, as for other gradient search or boosting approaches, there is no clear bound for the parameter *k*. Usually we need to carry out the iterations until a well-defined maximizer of the CV score to carry out the iterations until a well-defined maximizer of the CV score is found.

In a linear regression model, [7] shows that the TGDR can provide a path connecting the solutions roughly corresponding to the PLS/RR (ridge regression) and the solutions roughly corresponding to the LASSO by varying the thresholds. Moderate to large threshold values create paths that involve more diverse absolute coefficient values than the PLS/RR but less than the LASSO. Our numerical studies suggest that the conclusions drawn from the linear regression are applicable here.

As for other regularized variable selection approaches, we identify the best predictive model using cross validation. Usually in the final model, only a fraction of the estimated coefficients are nonzero, and those nonzero coefficients correspond to covariates importantly associated with the outcome. In certain variable selection approaches like the step-down, variable selection is achieved by excluding variables with marginal measurements like the p-values greater than certain cutoff. In the TGDR approach, variable selection is obtained by setting the coefficients corresponding to noisy variables zero. We can see from the algorithm description that there is no requirement on *d *for a given *n*. So when using the TGDR, we usually do not carry out a preliminary gene selection.

#### Tuning parameter selection

We use *V*-fold cross validation [33] to select the tuning parameter *k *for a given *τ*. For a pre-defined integer *V*, partition the dataset randomly into *V *non-overlapped subsets of equal sizes. Choose *k *to maximize the cross-validated objective function

$\text{CV score}=\sum_{v=1}^{V}{\hat{AUC}}_{normal}^{v}({\hat{\beta }}^{\left(-v\right)}),\;\left(5\right)$

where $\hat{\beta }$^(-*v*) ^is the proposed estimate of *β *based on the data without the *v*^*th *^subset for a fixed *k *and ${\hat{\text{AUC}}}_{normal}$ is the function ${\hat{AUC}}_{normal}^{\left(v\right)}$ defined in (3) evaluated with the *v*^*th *^subset only. Since the CV score defined in (5) involves computation of the binormal AUC, which measures the difference between two phenotype classes, the usual leave-one-out cross validation is not applicable here.

Since the performance of the TGDR estimates for different threshold values is of interest, we employ cross validation with respect to *k *only. In addition, cross validation over both *k *and *τ *will lead to models with slightly better prediction performance (than model achieved by cross validation over *k *only) but too many genes, hence unstable models. We have discussed the effect of different *τ *in the application. Related discussions can also be found in [34]. Beyond selecting the model (corresponding to the cross validated tuning parameters) with the best predictive performance, the *V*-fold cross validation also provides partial protection against overfitting [1].

The purpose of cross validation is to select the tuning parameters for estimating the parameter *β*. In practice, to evaluate how the model fit the data, the data set is usually divided into a training set and a test set (See the usage of this technique below in prediction performance evaluation). The parameter *β *is first estimated using the training set and the estimate is then applied to the test set for evaluation of the model. Thus the cross validation process is only used in the first step.

### Occurrence index

In "classic" classification studies, the standard approach for assessing the significance of a covariate is to use the p-value of its z-score, which is computed as the ratio of the estimated coefficient over its estimated standard error. However, when the sample size is smaller than or comparable to the number of covariates, this standard approach may not be appropriate, since its validity typically relies on the assumption *n *>> *d*.

In classification using microarray data, it is important to assess the relative importance and stability of genes, especially the genes identified to be correlated with the outcome. Motivated by the methods of [35,36], we propose the following measure for assessing the relative importance and stability.

1. We first generate perturbation of the data by randomly sampling *n*_1 _subjects. We propose *n*_1 _~ 2/3*n*.

2. The TGDR approach (including cross validation and evaluation) is then applied to the sampled subset. Repeat this procedure *B *(for example 1000) times.

3. For the *j*^*th *^covariate, compute the number of times *c*_*j *_it is included in the final model (the estimated coefficient is not zero) based on the *B *perturbed estimates. Then the proportion *o*_*j *_= *c*_*j*_/*B *gives a measure of the relative importance and stability of the *j*^*th*^covariate.

We call *o*_*j *_the occurrence index of the *j*^*th *^covariate. It lies between 0 and 1. We generate a large number of realizations of data with the same distribution as the observed data by partition. So loosely speaking, the occurrence index measures the stability of individual genes identified using the proposed approach. A higher occurrence index indicates a more stable and relatively more important gene.

The occurrence index shares the same spirit as the gene frequency in [22]. They both measure the stability of discovered genes using random sampling method. The essential difference is that in this paper, we consider joint models, i.e., the linear combinations of genes; whereas in [22] the marginal models are considered, i.e, the models with individual genes.

### Prediction performance evaluation

The following prediction performance evaluation is proposed in [11]. We refer to that paper for details. For completeness of this article, we briefly describe it below. The essence of the proposed approach is to evaluate prediction significance using randomly permuted and partitioned data.

#### Observed predictive distribution of AUC

1. We first partition the data randomly into a training set of size *n*_1 _and a testing set of size *n*_2 _with *n*_1 _+ n_2 _= *n*. Dudoit [20] suggests *n*_1 _~ 2/3*n*.

2. We use the training set to compute the ROC estimator. The binormal AUC for the testing set is then computed using this training set estimate.

3. To take into account the fact that we may get a large value of the AUC by chance with a "lucky" partition, we repeat this process many (for example 1000) times. Each time a new partition is made and the testing set AUC is computed.

With this procedure, we obtain a Monte Carlo estimation of the distribution of the prediction AUC by partitioning the observed data. We call it the *observed predictive distribution *(OPD) of AUC, which provides an honest measure of the classification performance of the proposed methodology. The OPD can be used to compare the relative performance of two approaches/estimates.

#### Permutation predictive distribution of AUC

We first randomly permute the binary outcome *Y*, but keep the indices of the covariates fixed. We then couple the permuted outcome with the covariates. We permute the data 1000 times. Each time, we partition the permuted data into a training set of size *n*_1 _and a testing set of size *n*_2_, and compute the testing set AUC in the same manner as described above. This yields the Monte Carlo distribution of the AUC with permuted data, i.e., the *permutation predictive distribution *(PPD) of AUC.

Well separated OPD and PPD distributions indicate that the model estimated with the proposed approach is effective in terms of prediction, whereas substantially overlapped distributions suggest that either the proposed approach is not effective or the biomarkers do not have good discriminant power.

We note that there are at least two possible approaches for evaluating prediction performance, one is based on cross-validation which randomly partitions the data into a training set and a test set, the other uses the bootstrap. Both approaches are equally valid. For detailed discussions, see Chapter 7 (in particular, Sections 7.10 and 7.11) of [32]. The approach we take is based on cross-validation, but beyond this, we used many random partitions to obtain the observed prediction distribution (OPD), and then compare it to the permutation prediction distribution (PPD), to evaluate the model significance in terms of prediction. According to [32], evaluation of prediction performance based on random partition or bootstrap are equally justified. In our case, we believe that random partition is more intuitive and computationally simpler. In the bootstrap approach, the standard bootstrap actually gives biased estimation, since it samples the data with replacement. Instead, one needs to use a "0.632 bootstrap" to avoid the bias and other subtle adjustments are needed, again, see [32].

## Authors' contributions

SM devised and implemented the procedure proposed in the paper, and drafted the manuscript. XS helped with the Normal ROC method and revising the manuscript. JH, together with SM, devised the approach for variable selection and prediction evaluation, helped with the TGDR algorithm, data analysis and revising the manuscript.
